# Exploring the molecular and immune landscape of cellular senescence in lung adenocarcinoma

**DOI:** 10.3389/fimmu.2024.1347770

**Published:** 2024-08-29

**Authors:** Kun Ru, Liang Cui, Cong Wu, Xin X. Tan, Wen T. An, Qiang Wu, Yu T. Ma, Yu Hao, Xiao Xiao, Jing Bai, Xiang Liu, Xue F. Xia, Miao Q. Zhao

**Affiliations:** ^1^ Department of Pathology, Shandong Cancer Hospital and Institute, Shandong First Medical University and Shandong Academy of Medical Sciences, Jinan, China; ^2^ Geneplus-Beijing Institute, Beijing, China; ^3^ Geneplus-Shenzhen Clinical Laboratory, Shenzhen, China; ^4^ Xiangya School of Pharmaceutical Sciences, Central South University, Changsha, China; ^5^ Key Laboratory Experimental Teratology of the Ministry of Education, Department of Physiology, School of Basic Medical Sciences, Cheeloo College of Medicine, Shandong University, Jinan, China; ^6^ School of Tropical Medicine and Laboratory Medicine, Hainan Medical University, Haikou, China; ^7^ Geneplus-Shenzhen Institute, Shenzhen, China; ^8^ Department of Thoracic Surgery, The Second Affiliated Hospital, Hengyang Medical School, University of South China, Hengyang, China

**Keywords:** cellular senescence, lung adenocarcinoma, tumor microenvironment, heterogeneity, machine learning

## Abstract

**Introduction:**

The connection between aging and cancer is complex. Previous research has highlighted the association between the aging process of lung adenocarcinoma (LUAD) cells and the immune response, yet there remains a gap in confirming this through single-cell data validation. Here, we aim to develop a novel aging-related prognostic model for LUAD, and verify the alterations in the genome and immune microenvironment linked to cellular senescence.

**Methods:**

We integrated a comprehensive collection of senescence genes from the GenAge and CellAge databases and employed the least absolute shrinkage and selection operator (LASSO) Cox analysis to construct and validate a novel prognostic model for LUAD. This model was then utilized to examine the relationship between aging, tumor somatic mutations, and immune cell infiltration. Additionally, we explored the heterogeneity of senescence and intercellular communication within the LUAD tumor microenvironment (TME) through single-cell transcriptomic data analysis.

**Results:**

By exploring the expression profiles of 586 cellular senescence-related genes in 428 LUAD patients, we constructed an aging-related genes (ARGs) risk model included 10 ARGs and validated it as an independent prognostic predictor for LUAD patients. Notably, patients with low aging scores (LAS group) exhibited better survival, lower tumor mutation burden (TMB), lower somatic mutation frequency, lower tumor proliferation rate, and an immune activated phenotype compared to patients with high aging scores (HAS group). While the HAS group was enriched in tumor cells and showed a lower infiltration of CD8-CCR7, CD8- CXCL13, CD8-GNLY, FCGR3A NK cells, XCL1 NK cells, plasma cell (PC) and other immune subsets. Furthermore, the SPP1 and TENASCIN pathways, associated with tumor immune escape and tumor progression, were also enriched in the HAS group. Additionally, our study also indicated that senescence levels were heterogeneous in the LUAD tumor microenvironment (TME), especially with tumor cells in the LAS group showing higher age scores compared to those in the HAS group.

**Conclusions:**

Collectively, our findings underscore that ARRS through ARGs serves as a robust biomarker for the prognosis in LUAD.

## 1 Introduction

Cancer with complex molecular characteristics (1), remains a significant global health challenge, accounting for a substantial number of deaths and impacting life expectancy worldwide. Amid the array of cancer types, lung cancer emerges as the second most prevalent contributor to cancer-related mortality, marked by a discouraging 5-year relative survival rate of just 23% (2). Lung adenocarcinoma (LUAD), the predominant histological subtype within non-small cell lung cancer, constitutes over 40% of all lung cancer cases (3). Notably, LUAD continues to rise in incidence among current smokers, former smokers, and even non-smokers, and its five-year survival rate remains dishearteningly low at approximately 15%, as a significant majority of patients are typically diagnosed at advanced stages of the disease (4). Hence, there is still a compelling need to formulate a novel prognostic model for predicting the outcomes of LUAD to advance more potent strategies for diagnosis and treatment.

Aging is a ubiquitous biological process that results in a progressive and irreversible decline in physical function across all organ systems, which presents with genomic instability, telomere attrition, epigenetic alterations, loss of proteostasis, disabled macroautophagy, deregulated nutrient-sensing, mitochondrial dysfunction, stem cell exhaustion, chronic inflammation, altered intercellular communication, cellular senescence, and dysbiosis (5–8). Cellular senescence refers to the essentially irreversible arrest of cell proliferation (growth) that occurs when cells experience potentially oncogenic stress (damage to DNA, strong mitogenic signals, damage or disruptions to the epigenome, and ectopic expression of certain tumor suppressors) (9, 10). Several evidences have shown that cellular senescence plays a double-edged role in initiation, growth, and progression of tumor (11, 12). Senescent tumor cells wield influence over the tumor microenvironment (TME) via the senescence-associated secretory phenotype (SASP). On one hand, by emitting pro-inflammatory cytokines, chemokines, growth factors, and proteases like IL-6, IL-8, and TGF-β, senescent cells can trigger paracrine senescence, transforming neighboring non-senescent cells into senescent counterparts. This process recruits and activates immune cells within the TME, leading to outcomes that can either hinder or foster tumor growth. M1 macrophages and natural killer cells, for instance, can eliminate tumor cells and foster their senescence through the secretion of IFN-γ and TNF-α, thereby restraining tumor expansion. On the other hand, senescent tumor cells may activate myeloid-derived suppressor cells and M2 macrophages via SASP, affecting the clearance of senescent tumor cells, in turn, driving tumor progression and vascularization (9, 13, 14). Given the role of cellular senescence in constraining tumor development, it emerges as a potential target for tumor therapy. Hence, unraveling the impact of senescence in tumorigenesis is paramount importance.

In recent years, several studies have focused on the role of senescence in LUAD (15–20). For example, Lin et al. constructed a cellular senescence-related signature (SRS) by leveraging senescence-related genes. They found that SRS involved in the regulation of the tumor immune microenvironment through SASP was a robust biomarker for the immunotherapeutic response and prognosis in LUAD (15). In a similar vein, another research by Lin et al. explored cellular senescence patterns within LUAD by analyzing mRNA expression profiles of 278 cellular senescence-related genes, demonstrating the association between cellular senescence patterns and tumor immune infiltration in LUAD (16). Besides, Liu et al. developed a 12-gene signature for LUAD using 91 cancer-related senescence genes to assess survival outcome (19). Nonetheless, prior investigations were marked by limitations. Firstly, all focused on only a subset of senescence genes. Secondly, the assessment of the TME was largely confined to the bulk transcriptomic level. As a result, the role of senescence in LUAD has yet to undergo systematic evaluation, and the intricate interplay between senescence and LUAD prognosis has remained obscure.

This current study seeks to overcome these limitations by integrating a comprehensive collection of 586 senescence genes sourced from the GenAge and CellAge databases. Employing the least absolute shrinkage and selection operator (LASSO) Cox analysis, a novel prognostic model for LUAD was constructed and validated. This model was further investigated the relationship between aging and tumor somatic mutation or immune cell infiltration. Furthermore, this study delved into the senescent heterogeneity and intercellular communication of various cells within the LUAD TME through the analysis of single-cell transcriptomic data. In summary, this study enriches our understanding of the profound impact of cell senescence on the survival outcomes of patients with LUAD, which unravels the complex associations between senescence, the immune landscape, and the intricate genetic makeup of the tumor, ultimately illuminating novel avenues for therapeutic interventions and prognostic assessments.

## 2 Materials and methods

### 2.1 Data source and processing

In the training cohort, bulk RNA sequencing (RNA-seq) data, somatic mutation data and clinical information for LUAD were downloaded from The Cancer Genome Atlas (TCGA) database (https://portal.gdc.cancer.gov/) (21). After excluding non-primary cases and patients with incomplete follow-up information, we analyzed 428 patients from the TCGA dataset as the training set. For the validation cohort (GSE31210, GSE50081, and GSE30219) (22–25), transcriptome data were obtained from data series in the Gene Expression Omnibus (GEO) database (https://www.ncbi.nlm.nih.gov/geo/) (26). Single-cell RNA-seq (scRNA-seq) data (GSE189357) comprising nine patients with LUAD was also download from the GEO database (27). Fragments per kilobase million (FPKMs) values or raw gene expression counts were normalized to transcripts per kilobase million (TPMs) in both the training and validation cohorts. Genes that were not expressed in more than half of the samples were excluded from the expression profiles. The clinical features of 428 patients are listed in **Table 1**.

**Table 1:** Patient characteristics for TCGA_LUAD cohort.

		Total (n = 428)	HAS group (n = 214)	LAS group (n = 214)	Fisher’s Exact Test (P value)
Age	<=65	206	111	95	0.142
	>65	212	98	114	
	NA	10	5	5	
Gender	female	238	111	127	0.144
	male	190	103	87	
race	american indian or alaska native	1	1	0	0.154
	asian	8	6	2	
	black or african american	47	19	28	
	white	330	168	162	
	NA	42	20	22	
OS	Alive	321	146	175	**0.002**
	Dead	107	68	39	
AJCC	I	245	105	140	**0.004**
	II	103	59	44	
	III	59	38	21	
	IV	14	9	5	
	NA	7	3	4	
T stage	T1	149	56	93	**0.002**
	T2	231	130	101	
	T3	36	22	14	
	T4	11	5	6	
	TX	1	1	0	
N stage	N0	292	130	162	**0.001**
	N1	77	47	30	
	N2	50	35	15	
	N3	2	1	1	
	NX	6	1	5	
	NA	1	0	1	
M stage	M0	286	147	139	0.313
	M1	14	9	5	
	MX	124	56	68	
	NA	4	2	2	

The “NA” represents sample with missing clinical information. Samples with missing clinical information were not considered in Fisher’s Exact Test statistics.

### 2.2 Aging gene set and screening

The set of aging-related marker genes (ARGs) was obtained from two databases, GenAge and CellAge. Initially, 279 ARGs were selected from CellAge (https://genomics.senescence.info/cells/) (28), and an additional 307 ARGs were obtained from GenAge (https://genomics.senescence.info/genes/index.html) (29) (**Supplementary Table S1**). Univariate Cox analysis was conducted by survival (version 3.3-1) packages to preliminarily identify ARGs associated with the overall survival (OS) of LUAD patients in the TCGA cohort (30), resulting in a final gene set comprising 102 ARGs (**Supplementary Table S2**).

### 2.3 Construction and validation of an ARGs risk model

We utilized the “glmnet” (version 4.1-8) package in R software (version 4.1.2) to perform the LASSO Cox regression analyses (family=“cox”) to screen out the prominent genes (31, 32). The “lambda.1se” value, determined through tenfold cross-validation, was employed as the lambda for model fitting. Ten genes were ultimately selected to construct the risk model. The prognostic capability of the ten genes was assessed using Kaplan-Meier survival curves generated with the survminer (version 0.4.9) and survival (version 3.3-1) R packages (30). Subsequently, we calculated a risk score for each sample, as a linear combination of gene expression levels within the signature set, weighted by their respective LASSO Cox regression coefficients, using the following formula:


$$\text{Aging-related risk scores }\left(\text{ARRSs}\right)=\sum_{i}^{n}Expre(gene_{i})*Coef\left(gene_{i}\right)\;$$

Here, “Coef (gene_i_)”, signifies the LASSO Cox regression coefficient, “Expre (gene_i_)”, represents the expression level of each gene, and “n” denotes the number of genes included in the model. In addition, the R package “survival” (version 3.3-1) was used to construct multiple multivariate Cox analysis to determine the independent prognostic factor in LUAD patients (30).

In the TCGA training cohort, LUAD patients were classified into high aging score group (HAS group) and low aging score group (LAS group) based on the median value of ARRSs. The prognostic capability of the risk model in terms of OS and progression-free survival (PFS) was assessed using Kaplan-Meier survival curves generated with the survminer (version 0.4.9) and survival (version 3.3-1) R packages (30). Additionally, we also compared the clinicopathological characteristics of TCGA-LUAD patients between the HAS group and the LAS group using Fisher’s Exact Test.

To validate the ARGs Risk Model, we calculated the risk score for patients in the validation cohort (GSE31210, GSE50081, and GSE30219) using the same formula as applied to the TCGA-LUAD cohort. Patients in the validation cohort were also categorized into high and low-risk groups based on the median value of ARRS. Kaplan-Meier curves were generated to assess the relationship between ARRS and OS in the validation cohort.

### 2.4 Functional enrichment analysis of differentially expressed genes based on HAS and LAS groups

We used the “DESeq2” (version 1.36.0) R package to calculate fold-changes and identify differentially expressed genes (DEGs) based on the two risk groups (false discovery rate (FDR) < 0.05 and |Log2FC| > 1) (33). Subsequently, we conducted Gene Ontology (GO) and Kyoto Encyclopedia of Genes and Genomes (KEGG) analyses on these DEGs using the “clusterProfiler” (version 4.7.1.002) R package (34). Pathways with adjusted p-values less than 0.05 were considered significant.

### 2.5 Immune infiltration between the HAS-group and LAS-group from TCGA-LUAD cohort

The “estimate” R package, a powerful tool for quantifying the immune stromal, and ESTIMATE scores, which was based on the expression of related molecular biomarkers in immune and stromal cells, to predict the TME (35). The “xCell” is a robust algorithm that analyzes the infiltration levels of 64 immune and stroma cell types, including extracellular matrix cells, epithelial cells, hematopoietic progenitors, innate and adaptive immune cells (36). Herein, we utilized the R package estimate (version 1.0.13) and xCell (version 1.1.0) to evaluate the immune infiltration score and immune cell infiltration in each patient between HAS and LAS subgroups. Additionally, the T cell-inflamed gene expression profile (GEP) was calculated as a weighted sum of standardized expression values of 18 genes (*CCL5, CD27*, *CD274*, *CD276*, *CD8A*, *CMKLR1*, *CXCL9*, *CXCR6*, *HLA-DQA1*, *HLA-DRB1*, *HLA-E*, *IDO1*, *LAG3*, *NKG7*, *PDCD1LG2*, *PSMB10*, *STAT1*, *TIGIT*) as described in previous literature (37–39). The single sample gene set enrichment analysis (ssGSEA) algorithm in “gsva” (version 1.42.0) R package was performed to compare differences in 13 gene sets associated immune function and 4 gene sets related to angiogenesis, matrix, matrix remodeling, and tumor proliferation rate from previous studies (40–42). Box plots were developed using ggplot2 software (version 3.4.3) in R to display the differences between the two groups (43).

### 2.6 The genetic landscapes of HAS-group and LAS-group

Genetic landscapes were analyzed and visualized using the “maftools” (version 2.12.0) R package (44). Tumor Mutation Burden (TMB) was defined as the number of somatic, non-silent, protein-coding mutations in the coding regions per megabase (mut/Mb) and counted using ‘maftools’ (version 2.12.0). The mutated samples of tumor-related and DNA damage repair (DDR) pathways in HAS and LAS groups were compared using Fisher’s exact test (with p <0.05 indicates a significant difference) and visualized using “ggradar” (version 2.12.0) and ggplot2 (version 3.4.3) R packages (43).

### 2.7 Single-cell RNA-seq analysis

Raw matrix data were obtained from the GEO database for subsequent analysis (27). Initially, cells with low quality were filtered out based on the following criteria: 1) fewer than 200 expressed genes, 2) total molecule count per cell less than 800, and 3) greater than 10% of reads mapped to the mitochondrial genome. Additionally, the “DoubletFinder” R package (45) was utilized to identify and remove doublet cells using default parameters.

The “Seurat” package (version 4.3.0) (46) was employed to normalize the single-cell gene expression data using the “NormalizeData” and “ScaleData” functions, respectively. Subsequently, the top 2,000 highly variable genes for each sample were selected using the “FindVariableFeatures” function. Principal component analysis (PCA) was performed using the “RunPCA” function, and the first 20 principal components were used for Uniform Manifold Approximation and Projection (UMAP) analysis with the “RunUMAP” function. Following UMAP analysis, cells were clustered using an unsupervised method with a resolution parameter of 1.5 employing the “FindNeighbors” function. Differential expression analysis was conducted on the original log-normalized data by comparing cells within each cluster to all other clusters using the “FindAllMarkers” function. Clusters were annotated based on the expression of well-known markers and differentially highly expressed genes.

Subgroup analysis of each cell group, including T/NK cells, B cells, and myeloid cells, was performed using the standard Seurat pipeline. Specific markers were used for grouping and are listed in **Supplementary Table S3**. Bar plots were generated to illustrate the percentage of cells between the two groups. Additionally, cell occupancy differences were assessed using Fisher’s exact test. The cytotoxic and exhausted scores for T cell subgroups, as well as the hallmark pathways compared between HAS and LAS groups, were calculated using the ssGSEA algorithm in the “gsva” package (version 1.42.0) based on different sets of genes (42).

### 2.8 Identification of cancer cells

To identify cancer cells, we utilized the inferCNV (version 1.13.0) tool (https://github.com/broadinstitute/inferCNV), as previously described in studies by Liu, He, et al. and Chen et al. (47, 48). The inferCNV package compares gene expression profiles of each cell to reference gene expression profiles from other cells. Initially, raw count data and cell type annotations for all cells were extracted from the Seurat object. Immune cells and stromal cells were chosen as reference cells. A gene ordering file was generated from the human GRCh38 assembly, containing chromosomal start and end positions for each gene. These files were used to create an inferCNV object using the “CreateInfercnvObject” function, followed by running inferCNV with default parameters. The calculated copy number variation (CNV) signal was defined as the mean square of CNV estimates across all genomic locations. CNV R-scores were calculated as the Pearson correlation coefficient between each cell’s CNV pattern and the average CNV pattern of the top 5% of cells from the same tumor based on CNV signal. Cells with CNV R-scores ≥0.3 were classified as tumor cells.

### 2.9 Aging-related risk scores based on pseudo-bulks

The Seurat object was transformed into a “SingleCellExperiment” object, followed by the computation of pseudo-bulks. Pseudo-bulks, which represent the sum of counts, were calculated using aggregation-based methods in the muscat (version 1.10.1) R package (https://github.com/HelenaLC/muscat). The ARRSs were then derived using the previously described formula based on the pseudo-bulks. Patients were stratified into two groups, HAS and LAS, based on the median value of ARRSs. Additionally, age scores for each cell were calculated based on ten ARKGs at the single-cell level using the ssGSEA algorithm.

### 2.10 Cell-cell interactions

CellChat (version 1.5.0) is an open-source R package (https://github.com/sqjin/CellChat) utilized for the analysis, comparison, and visualization of single-cell RNA sequencing data intercellular communication (49). In this study, CellChat was employed to infer cell-cell interactions across 24 immune subgroups, fibroblasts, normal epithelial cells, tumor cells, and endothelial cells for both the HAS and LAS groups. Subsequently, major signaling changes between the HAS and LAS groups were computed.

### 2.11 Statistical analysis

The Wilcoxon test was conducted to examine differences in variables between two groups, while the Kruskal-Wallis test was used to assess differences among groups greater than two. Gene mutation differences between the HAS and LAS groups were determined using Fisher’s exact test.

## 3 Results

### 3.1 Construction and validation of aging-related risk score

**Figure 1:**
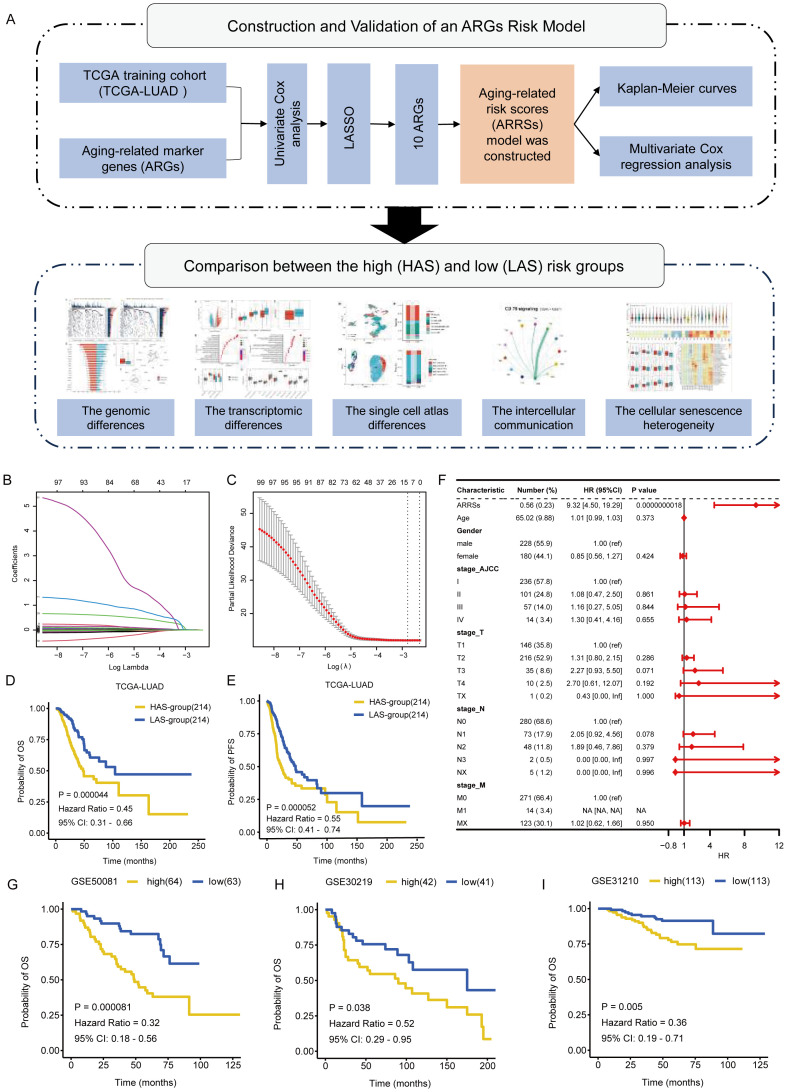
Identification of ARKGs related to prognosis in the TCGA training cohort. **(A)** The workflow of the present study. **(B)** Selection of optimal candidate genes in the LASSO model. **(C)** LASSO coefficients of prognosis-associated ARKGs. **(D, E)** Kaplan-Meier curves for overall survival **(D)** and progression free survival **(E)** of the TCGA-LUAD cohort in the HAS and LAS groups. **(F)** Forest plots showing results of multivariate Cox regression analysis between Risk score, clinical information and overall survival. **(G-I)** Kaplan-Meier curves for overall survival of validation cohorts in the high and low groups: GSE50081 **(G)**, GSE30219 **(H)**, GSE31210 **(I)**.

The workflow of the whole study was graphically presented in **Figure 1A**. We compiled a comprehensive list of 586 aging-associated genes sourced from the CellAge and GenAge databases. Among these genes, 102 were significantly associated with clinical survival (p < 0.05) based on univariate Cox analysis (detailed results shown in **Supplementary Table S2**), conducted on the expression matrix and clinical survival information of 428 LUAD samples obtained from the TCGA dataset. Subsequently, to construct the ARGs risk model, we performed LASSO Cox regression analysis on the aforementioned 102 genes and the gene expression profiles of the training cohort (**Figures 1B, C**). Through this analysis, we successfully identified 10 aging-related key genes (ARKGs), including. *BRCA2*, *CSNK1E*, *EEF1E1*, *GAPDH*, *IGFBP3*, *IL1A*, *PSEN1*, *XRCC5*, *XRCC6*, and *YWHAZ*. And low RNA expression for the 10 ARKGS was correlated with longer survival time in LUAD (**Supplementary Figure S1**). Utilizing these ten ARKGs and their corresponding risk coefficients, we established an aging risk signature. The risk score of every patient was calculated using this formula. Patients in the training cohort were stratified into two groups: the high aging score group (HAS group) and the low aging score group (LAS group) based on median values of ARRSs. Upon investigating the expression levels of the ten ARKGs, we found that they were significantly higher in HAS group than LAS group (**Supplementary Figure S2**, **Supplementary Table S4**).

We compared the clinicopathological characteristics, including age, gender, race, OS, TNM tumor grade, and AJCC tumor grade, of TCGA-LUAD patients between the HAS group and the LAS group (**Table 1**). The results showed significant differences in OS status (P = 0.002), T grade (P = 0.002), N grade (P = 0.001), and AJCC tumor grade (P = 0.004) between the groups. Survival analysis demonstrated that the LAS-group exhibited significantly improved overall survival (OS) (hazard ratio (HR) = 0.45, 95% confidence interval (CI) = 0.31 – 0.66, P = 0.000044) and progression-free survival (PFS) (HR =55, 95% CI = 0.41 – 0.74, P = 0.000052) compared to the HAS-group (**Figures 1D, E**). Upon integrating age, gender, TNM tumor grade, and AJCC tumor grade into the multivariate Cox regression analysis, ARRSs emerged as the sole significant survival-related risk factor (HR = 9.32, 95% CI = 4.50 – 19.29, P = 0.0000000018) (**Figure 1F**), suggesting that ARRSs was an independent prognostic factor for LUAD.

To validate the prognostic roles of the above risk model, we applied the same stratification method to three independent datasets form the GEO database. Consistent with the findings from the training cohort, patients with in the high ARRSs group displayed significantly worse survival outcomes compared with the low ARRSs group in all three cohorts, namely GSE50081 (HR = 0.32, 95% CI = 0.18 – 0.56, P = 0.000081), GSE30219 (HR = 0.52, 95% CI = 0.29 – 0.95, P = 0.038), and GSE31210 (HR = 0.36, 95% CI = 0.19 – 0.71, P = 0.005) (**Figures 1G-I**).

### 3.2 The genetic characteristics of HAS-group and LAS-group

**Figure 2:**
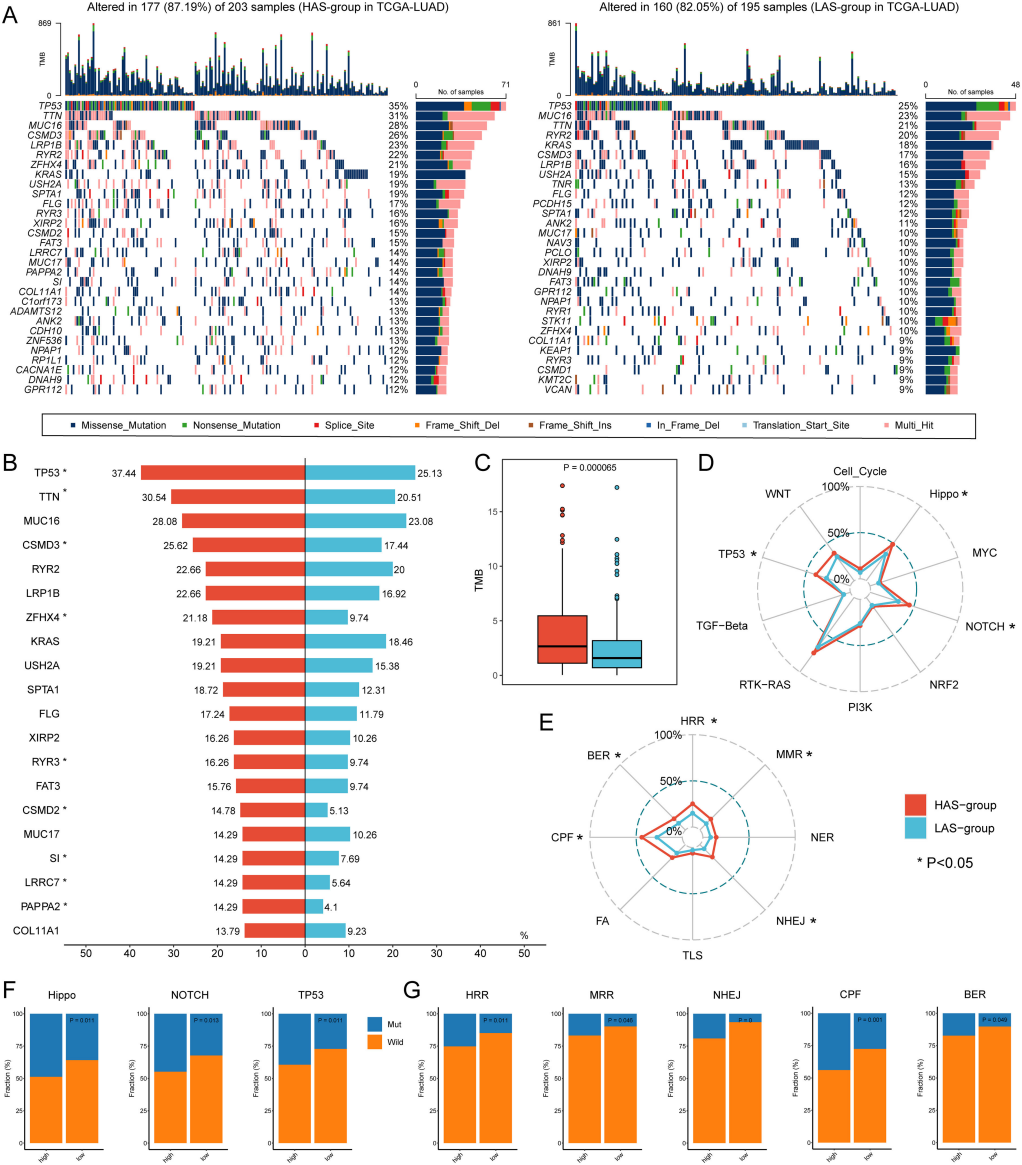
Genomic alterations differences between the HAS and LAS group from the TCGA-LUAD cohort. **(A)** Genomic alterations landscape between the HAS (left) and LAS (right) group. **(B)** Mutation frequency differences of the top 20 mutation genes in the HAS group compared to the LAS group. The asterisk to the right of the gene indicates that the mutations in the gene were significantly different in the two groups, as determined by Fisher’s exact test. **(C)** The TMB between HAS and LAS groups. The HAS group had a higher TMB (2.66 (IQR: 0.04, 11.985) compared to the LAS group (1.58 (IQR: 0.02, 6.9)) with P value = 0.000065 compared by the Wilcoxon test. The frequency of mutated genes in each tumor-related pathway **(D, F)** and DDR pathway **(E, G)** difference between two groups. The asterisks in **(D, E)** denote significant differences of mutated genes in different pathways identified by Fisher’s exact test which showed in **(F, G)**.

To explore the genetic features in LUAD with different ARRSs, we further investigated the genomic differences between the HAS group and the LAS group based on somatic mutation data in the TCGA-LUAD cohort (**Figure 2A**; **Supplementary Figures S3A-C**). We observed that HAS group had a higher mutation frequency than the LAS group, particularly in the top 20 genes such as, *TP53*, *TTN*, *CSMD3*, *ZFHX4*, *RYR3*, *CSMD2*, *SI*, *LRRC7*, and *PAPPA2* (detailed P values shown in **Supplementary Table S5**) between HAS and LAS groups (**Figure 2B**). Additionally, the HAS group displayed a higher tumor mutation burden (TMB) but a lower occurrence of co-occurring mutations between genes, indicating distinct genomic alteration patterns (**Figure 2C**; **Supplementary Figure S3D**). Further analysis of ten tumor-related pathways revealed significantly higher mutation frequencies in the Hippo (P = 0.011), NOTCH (P = 0.013), and TP53 (P = 0.011) pathways in the HAS-group compared to the LAS-group (**Figures 2D**, **F**; **Supplementary Figure S4B**). Similarly, higher mutation rates were observed in the HAS group among the eight DDR pathways, with five of them being statistically significant (**Figures 2E, G**; **Supplementary Figure S4A**).

### 3.3 ARRSs is associated with cell proliferation and immune function

**Figure 3:**
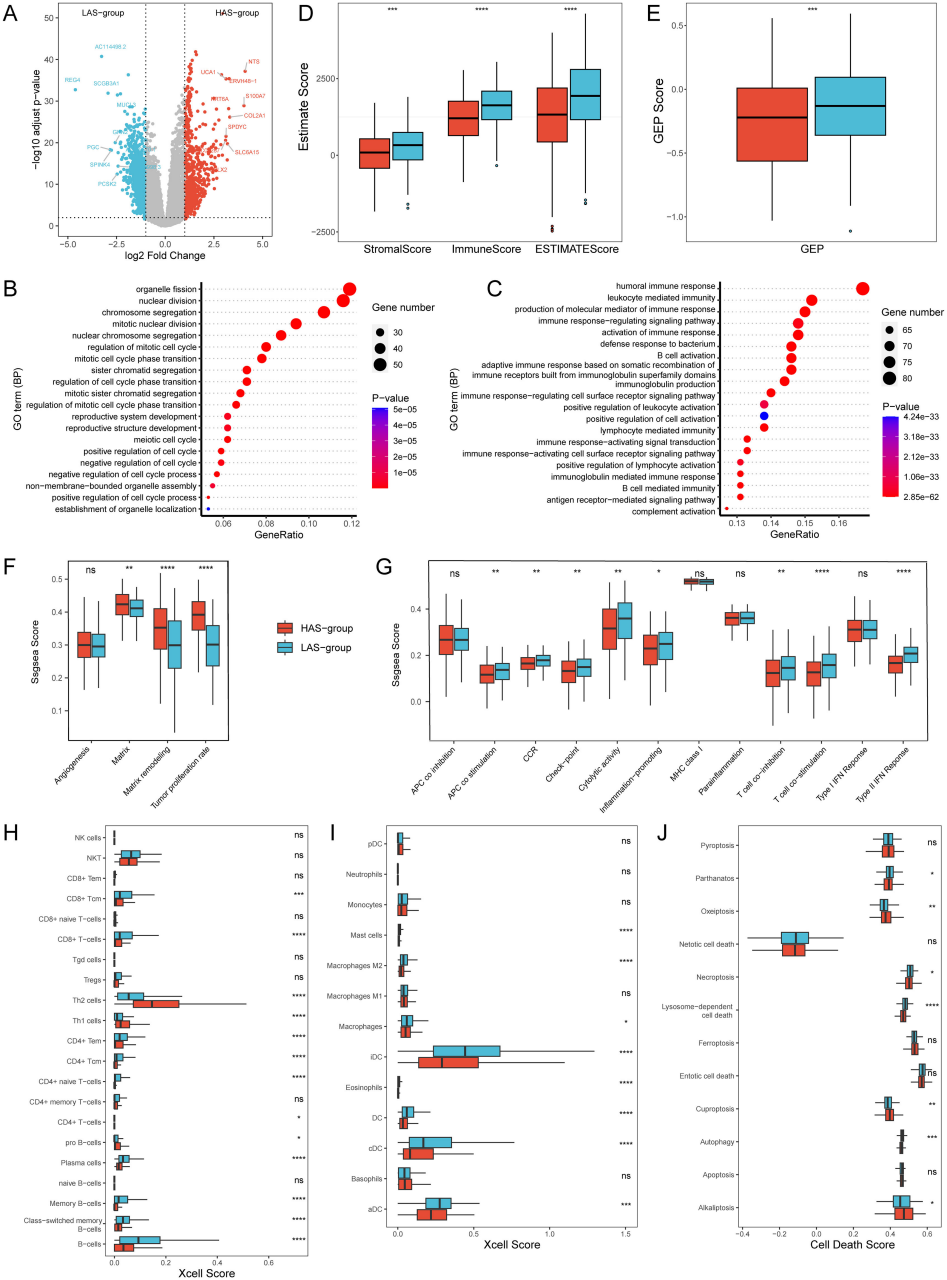
Transcriptomic differences between the HAS and LAS group from the TCGA-LUAD cohort. **(A)** Volcano Plot of DEGs between the HAS and LAS group. **(B, C)** Top 20 biological processes of GO enrichment results between the HAS **(B)** and LAS **(C)** group. **(D)** Stromal score, immune score and ESTIMATE score between the two groups. **(E)** GEP score between the two groups. **(F, G)** Boxplots of gene sets related to tumor proliferation **(F)** and immune-related functions **(G)**. **(H, I)** Box plot of T cells **(H)**, B cells **(H)**, and myeloid cells **(I)** infiltration in “Xcell” between the two groups. **(J)** Box plot of cell death between the two groups. "ns" indicates p > 0.05, * indicates p ≤ 0.05, ** indicates p ≤ 0.01, *** indicates p ≤ 0.001, and **** indicates p ≤ 0.0001. The actual P determined by the Wilcoxon test, and the medians (IQR) in **Figures 2D-F** were all displayed in **Supplementary Table S4**. All abbreviations presented in **Figure 3** showed as following: GEP, T cell-inflamed gene expression profile; CCR, cytokine and cytokine receptor; HLA, human leukocyte antigen; MHC, major histocompatibility complex.

Differential expression analysis of gene expression data based on the HAS group and LAS group identified a total of 1664 differentially expressed genes (DEGs) under a threshold of adjusted p < 0.05, comprising 707 up-regulated and 957 down-regulated genes (**Figure 3A**). GO enrichment analysis for DEGs revealed that in the HAS-group, biological processes were predominantly enriched in cell cycle, cell division, and cell development, indicating a potential involvement in regulating normal cell function and organismal development (**Figure 3B**). Furthermore, based on gene sets from Bagaev, et al. (40), we found that the tumor proliferation rate, and matrix remodeling of the HAS group were significantly higher than those of the LAS group (**Figure 3F**, detailed P values were shown in the **Supplementary Table S4**).

In contrast, the LAS-group exhibited enrichment in immune response mechanisms, encompassing cell activation, signal transduction, and production of immune mediators (**Figure 3C**). Based on another gene set related to immune functions (41), we observed that nine of the 13 immune function gene sets had significantly higher ssGSEA scores in all LAS groups than the HAS group (**Figure 3G**, detailed P values were shown in the **Supplementary Table S4**), especially type II IFN response, T cell co-stimulation, and HLA. Immune estimations for LUAD patients within the training set (TCGA-LUAD) showed notably increased StromalScore, ImmuneScore, ESTIMATEScore, and GEP score in the LAS group when compared to the HAS group (**Figures 3D, E**). Xcell analysis revealed the immune infiltration of TME (36). The results indicated that LAS group had an activated TME, with significantly increased numbers of T cells, such as CD8+ T cells, CD8+ Tcm, CD4+ Tem, and CD4+ Tcm, and significantly decreased numbers of Th1 and Th2 (**Figure 3H**, detailed p values were shown in the **Supplementary Table S4**). Additionally, B cells such as plasma cells (**Figure 3H**), and myeloid cells such as Mast cells, and various DCs (**Figure 3I**, detailed p values were shown in the **Supplementary Table S4**), were also significantly increased in the LAS group. Furthermore, we explored the relationship between ARRSs and various cell death pathways. The findings revealed that significantly elevated scores for Alkaliptosis, Cuproptosis, and Oxeiptosis in the HAS-group, whereas Autophagy, Lysosome-dependent cell death, Necroptosis, and Parthanatos scores were markedly higher in the LAS-group (**Figure 3J**; **Supplementary Table S4**).

### 3.4 The single cell alta of HAS-group and LAS-group

**Figure 4:**
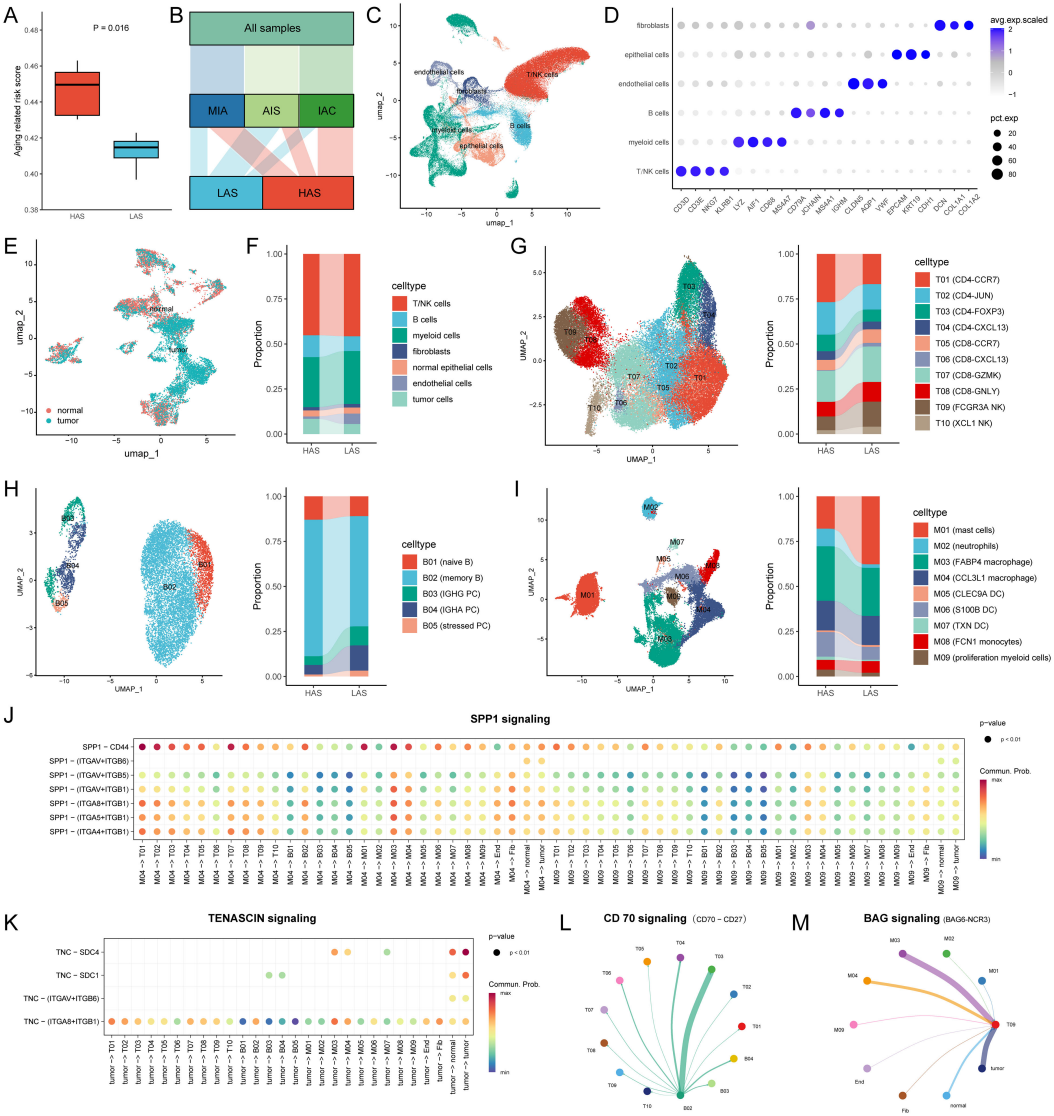
Single cell atlas and cell-cell interactions between the HAS (n = 5) and LAS (n = 4) group. **(A)** ARRSs based on single cell pseudo-bulks differed between the HAS and LAS group. The HAS group had a higher ARRS 0.4496 (IQR: 0.4304, 0.463) compared to the LAS group 0.4147 (IQR: 0.3969, 0.4228) with P value = 0.0159 compared by the Wilcoxon test. **(B)** Alluvial diagram showed the grouping of the nine samples. **(C)** UMAP plot for cells displaying the six major cell types from patients. **(D)** Dot plot depicting mean expression levels and percentage of cells expressing signature genes across the six major cell types. **(E)** Distribution of normal and tumor cells in epithelial cells from LUAD. **(F)** The composition of the cell compartment, displaying the average frequencies of cell subsets for HAS and LSA groups. **(G-I)** The UMAP plot and the average frequencies of different T cell, B cell and myeloid cell subgroups. **(J, K)** Comparison of the significant ligand-receptor pairs of SPP1 signaling **(J)** and TENASCIN signaling **(K)** for the HAS group. Dot color reflects communication probabilities and dot size represents computed p-values. Empty space means the communication probability is zero. p-values are computed from one-sided permutation test. **(L, M)** Circle plot showed cell–cell communication mediated by CD70-CD27 **(L)** and BAG6-NCR3 **(M)** in the LAS group. All abbreviations presented in **Figure 4** showed as following: ARRS, aging related risk score; IQR, interquartile range; AIS, lung adenocarcinoma *in situ*; MIA, minimally invasive adenocarcinoma; IAC, invasive adenocarcinoma; tumor, tumor cells; normal, normal epithelial cells; Fib, fibroblasts; End, endothelial cells.

To further investigate whether the ARRSs is heterogeneous in the TME, we utilized a single-cell dataset (GSE189357) containing over 10,000 cells from 9 patients. Initially, the single-cell dataset was converted to pseudo-bulks and then ARRSs were calculated. Subsequently, the 9 patients were divided into HAS (n = 5) and LAS (n = 4) groups based on the median value of ARRSs. Notably, two of the three invasive adenocarcinoma (IAC) samples were categorized into the HAS group, exhibiting significantly higher aging scores compared to the LAS group (**Figures 4A, B**). Employing the standard pipeline in Seurat (46), we identified six major cell types, including T/NK cells, B cells, myeloid cells, fibroblasts, endothelial cells, and epithelial cells (**Figures 4C, D**). Subsequently, the epithelial cells were further subdivided into tumor cells and normal epithelial cells (**Figure 4E**). Interestingly, we observed an enrichment of tumor cells and endothelial cells in the HAS (P = 6.44E-66, odds ratio (95% CI) = 1.57 (1.49, 1.66), **Supplementary Table S6**) and LAS (p = 0, odds ratio (95% CI) = 4.56 (4.2, 4.95)) groups, respectively (**Figure 4F**).

Furthermore, we conducted subtype annotation specifically for immune cells including T/NK cells, B cells, and myeloid cells (**Figures 4G-I**; **Supplementary Table S2**). T/NK cells were subdivided into eight T cell subpopulations and two NK cell subpopulations (**Figure 4G**). Functional scoring of T-cell subsets revealed that FCGR3A NK cells (T09) and CD8-GNLY (T08) had the highest cytotoxic scores, while CD8-CXCL13 (T06) had the highest exhausted score (**Supplementary Figure S5**). We compared the cellular infiltration in the HAS and LAS groups and found that the T and NK cell subpopulations were significantly differed between the HAS and LAS groups (**Supplementary Table S6**). Specifically, CD4-CCR7 (T01, P = 1.53E-134, odds ratio (95% CI) = 1.81 (1.72, 1.9)), and CD4-FOXP3 (T03, P = 4.55E-21, odds ratio (95% CI) = 1.41 (1.31, 1.52)) were enriched in the HAS group, whereas CD8-CCR7 (T05, P = 6.42E-14, odds ratio (95% CI) = 1.34 (1.24, 1.45)), CD8-CXCL13 (T06, P = 2.35E-64, odds ratio (95% CI) = 5.66 (4.51, 7.16)), CD8-GNLY (T08, P = 2.94E-23, odds ratio (95% CI) = 1.39 (1.3, 1.48)), FCGR3A NK cells (T09, P = 4.29E-98, odds ratio (95% CI) = 1.95 (1.83, 2.08)), and XCL1 NK cells (T10, P = 2.65E-34, odds ratio (95% CI) = 2.02 (1.8, 2.27)) were enriched in the LAS group. For B cell subsets, naive and memory B cells were more prevalent in the HAS group, whereas plasma cell (PC) subsets (B03 P = 1.33E-24, odds ratio (95% CI) = 2.3 (1.96, 2.69); B04, P = 3.51E-48, odds ratio (95% CI) = 2.96 (2.55, 3.43)) and stressed PC (B05, P = 7.24E-13, odds ratio (95% CI) = 3.02 (2.21, 4.12)) were more prevalent in the LAS group. The Mast cells (M01, P = 7.44E-292, odds ratio (95% CI) = 2.77 (2.62, 2.92)) showed a tendency to increase in the LAS group compared to the HAS group, while neutrophils (M02, P = 4.84E-164, odds ratio (95% CI) = 5.25 (4.55, 6.08)), S100B DC (M06, P = 1.08E-66, odds ratio (95% CI) = 2.05 (1.88, 2.23)), TXN DC (M07, P = 9.61E-15, odds ratio (95% CI) = 2.47 (1.93, 3.19)), and proliferation myeloid cells (M09, P = 1.51E-16, odds ratio (95% CI) = 1.87 (1.6, 2.2)) were significantly more prevalent in the HAS group. These results provide further evidence of heterogeneity in immune cell infiltration between groups with differing ARRs at the single-cell level, especially the LAS enriched more cytotoxic T/NK cells and antibody-secreting B cells.

### 3.5 Inference of cell-cell interactions

Given that senescence alters intercellular communication, we conducted a comparative analysis of intercellular communication between the HAS and LAS groups for each cell subset based on single-cell data. Significant differences were observed in several signaling networks between the HAS and LAS group (**Supplementary Figure S6**). Notably, SPP1 was exclusively present in in the HAS group (**Supplementary Figure S6**; **Figure 4J**). Especially, the interaction of SPP1-CD44 has been reported to inhibit T-cell activation and promote tumor immune evasion (50, 51). Additionally, TENASCIN was frequently observed in the HAS group, with tumor cells in this group interacting with other cells, including tumor cells themselves, via TNC - SDC1/SDC4 or TNC - ITGA8_ITGB1/ITGAV_ITGB6 (**Supplementary Figure S6**; **Figure 4K**). TNC is an extracellular matrix glycoprotein known to contribute to tumor progression, and increased TNC expression in LUAD tissues correlates with an unfavorable clinical outcome for patients (52). Conversely, certain pathways were exclusively or more frequently observed in the LAS group (**Supplementary Figure S6**). For example, the secreted signaling BAG, and CD70 pathways were uniquely found in the LAS group (**Supplementary Figure S6**). The BAG6-NCR3 interaction targeting T09 might trigger NK cell cytotoxicity (**Figure 4L**). Furthermore CD70-CD27 interaction was observed between B02 and PC or between B02 and T cells. CD27 receptor activation provides a costimulatory signal promoting T cell and B cell activity to enhance anti-tumor and anti-infection immunity (**Figure 4M**) (53).

### 3.6 Cellular senescence heterogeneity in the tumor microenvironment

**Figure 5:**
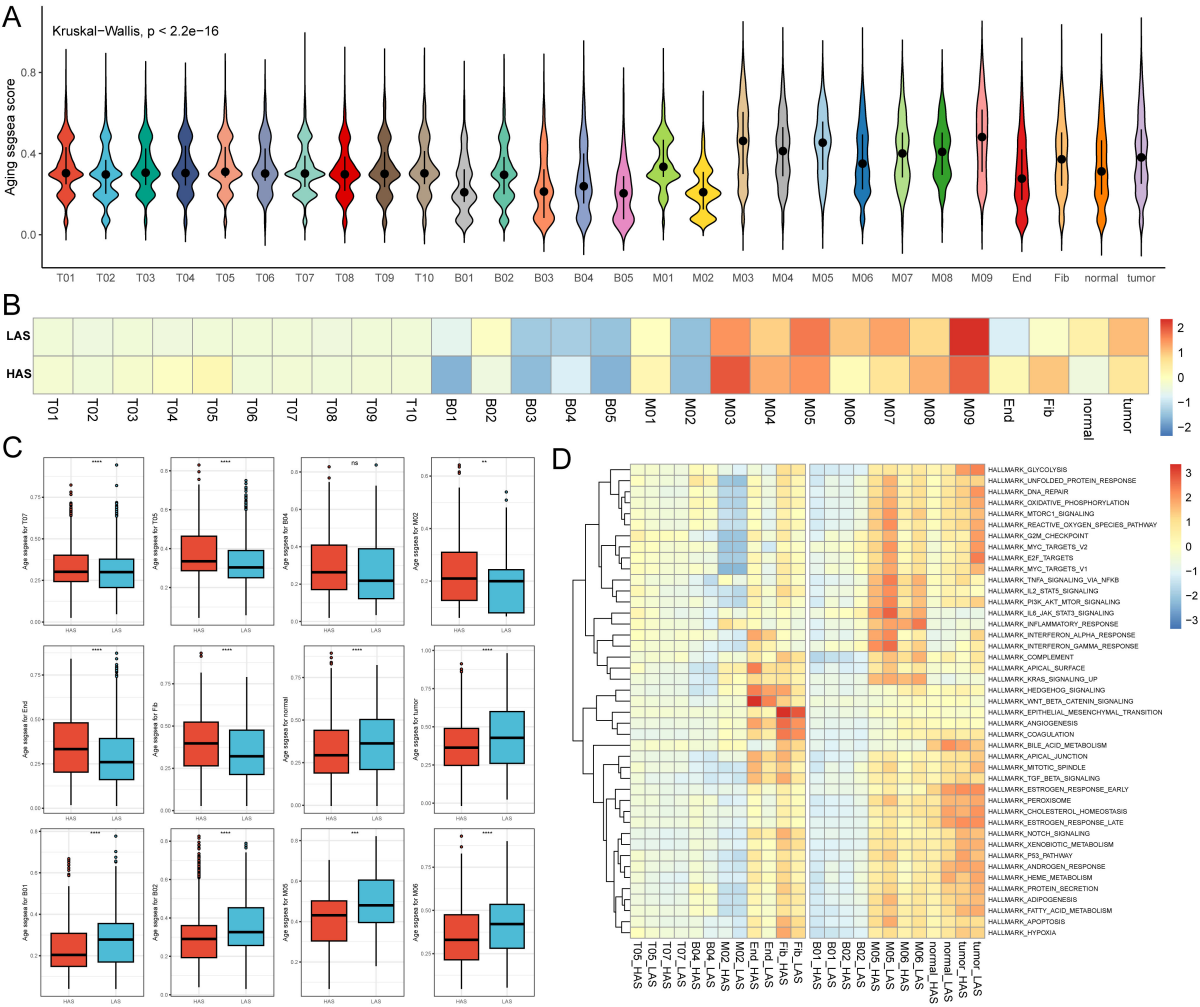
Single cell age score. **(A)** The age score for each cell. **(B)** The medians of age scores for each cell type in the HAS and LAS groups. **(C)** The box plots demonstrating between-group differences in the HAS and LAS groups for age scores for specific cell types. We used the following notation for statistical significance: “ns” indicates p > 0.05, ** indicates p ≤ 0.01, *** indicates p ≤ 0.001, and **** indicates p ≤ 0.0001. The actual P determined by the Wilcoxon test, and the medians (IQR) in **(C)** were all displayed in **Supplementary Table S4**. **(D)** Heatmap showed the activity of hallmarks between the HAS and the LAS groups for different cell types. All abbreviations presented in **Figure 5** showed as following: tumor, tumor cells; normal, normal epithelial cells; Fib, fibroblasts; End, endothelial cells.

Using single-cell data, we evaluated the senescence levels of individual cells and compared the senescence levels among different cell subpopulations (**Figure 5A**). We observed lower age scores in B01, B03, B05, M02, and endothelial cell subpopulations, while M03 and M05 exhibited higher age scores (**Figure 5A**). Subsequently, we compared the senescence levels of cell subpopulations between the HAS and LAS groups (**Figure 5B**). Most T cell subsets (e.g., T05, T07) displayed higher age scores in the HAS group than in the LAS group (**Figures 5B, C**). Moreover, endothelial and fibroblast cells exhibited higher age scores in the HAS group, whereas normal epithelial cells and tumor cells showed higher age scores in the LAS group (**Figures 5B, C**). Age scores for different subpopulations of myeloid and B cells varied between the HAS and LAS groups (**Figures 5B, C**). For instance, the age scores of B01, B02, M05 and M06 were significantly lower in the HAS group than in the LAS group, while B04 and M02 showed higher scores in the HAS group (**Figures 5B, C**). As cellular damage caused by reactive oxygen species (ROS) is a major trigger for senescence (54), we assessed and compared the “reactive oxygen species pathway”. Our results revealed higher scores for this pathway in the HAS group for B01, B02, M05, M06, normal epithelial cells, and tumor cells, whereas the HAS group for B04, M02, T05, T07, endothelial, and fibroblast cells exhibited lower scores (**Figure 5D**), consistent with the trend observed in age scores (**Figure 5C**).

## 4 Discussion

Cellular senescence involves the cessation of cell-cycle and the release of inflammatory cytokines with autocrine, paracrine and endocrine activities (55). The SASP represents a significant feature of senescent cells, encompassing the release of various cytokines, chemokines, growth factors and proteases (56). The impact of cellular senescence on cancer is intricate, displaying both advantageous and detrimental effects. Nevertheless, the extent to which the senescent heterogeneity of immune infiltration cells within tumors, as well as the interplay between tumor senescence and immune infiltration in LUAD, remains incompletely documented. In the current investigation, we constructed an independent prognostic model based on cellular senescence-related genes, and comprehensively analyzed the role of aging in genomic alterations and immune landscape in LUAD, which might hold the potential to facilitate the development of personalized immunotherapy.

This study successfully identified a novel and independent prognostic risk model incorporating ten significantly upregulated genes in LUAD. Ten genes were selected from a comprehensive list of 586 aging-associated genes obtained from the CellAge and GenAge databases. These genes also have been previously reported as positive regulators of tumor development. For example, *CSNK1E*, a member of the serine/threonine protein kinase family, controls circadian rhythms, which is closely related to the animals longevity (57). Inhibition of *CSNK1E* has been show to selectively inhibit tumor cell development (58), and elevated *CSNK1E* expression is associated with poor prognosis in patients with ovarian cancer and malignant melanoma (59, 60). *EEF1E1*, a tumor suppressor, plays a role in ATM/ATR-mediated p53 activation (61), and serves as a poor prognosis predictor in lung cancer (62). Overexpression of *EEF1E1* in transgenic mice resulted in a significantly shorter mean lifespan (63). *GAPDH* directly participates in tumor progression, invasiveness, and metastasis (64), and conditions such as oxidative stress impair *GAPDH* catalytic activity, leading to cellular aging and apoptosis (65). Increased expression of *PSEN1* in colorectal cancer is associated with enhanced tumor development through heightened EGFR signaling via NOTCH1 processing and activation of the COX-2-PGE2 pathway (66). PSEN1-null mice die shortly after birth (67), although *PSEN1*’s role in human aging remains largely unknown. *YWHAZ* is an adapter protein implicated in several signal transduction pathways (68) and interacts with numerous proteins associated with aging, such as the INS/IGF1 pathway (69, 70). *YWHAZ* has also been shown to mediate lung cancer malignancy and β-catenin protein through its complex with β-catenin (71). *IL1A*, a pivotal inflammatory cytokine, is thought to be one of the critical upstream regulators of other SASP-related genes (72, 73) and drives tumor growth and metastasis (74). *IGFBP3*, a member of the insulin-like growth factor-binding protein (IGFBP) family, regulates IGF1 and IGF2 by altering the interaction of IGFs with their cell surface receptors. Interestingly, the cell growth regulator *IGFBP3* exhibits a unique pattern, as elevated levels are associated with a good prognosis in patients with advanced NSCLC (75). *BRCA2*, *XRCC5*, and *XRCC6* are all DDR related genes, involved in DNA damage and repair. Mice deficient for *BRCA2* and *XRCC5* have a reduced lifespan (76, 77). *XRCC5/6* are associated with poor prognosis and can be used as diagnostic and prognostic biomarkers for LUAD (78). *BRCA2*’s role in cancer well-established, as elevated *BRCA2* expression is associated with a significantly reduced number of stromal cells and high infiltration of both beneficial and detrimental immune cells in breast cancer (79). *BRCA2* has also been demonstrated to exhibit increased mRNA levels and poor prognosis in lung cancer (80). These findings collectively provide compelling evidence that this newly proposed prognostic risk model has the potential to reflect LUAD prognosis by considering genomic alterations and the immune landscape.

Genetic instability is a common characteristic of both aging and cancer (81), encompassing changes in DNA damage, DNA damage response and repair, mutations, replication stress, transposition, chromosome aberrations, telomere shortening, micronuclei, and DNA fragments (82). In our study, we found that the HAS group exhibited more frequent gene mutations and higher TMB, indication the presence of an unstable genome and immunogenic potential in patients with HAS. Furthermore, the mutation frequency of the Hippo, NOTCH, TP53, and DDR pathways in the HAS group were also significantly increased. Hippo is an important pathway regulating differentiation, stem cell renewal, and oncogenic transformation (83). In cancer research, the activated Hippo pathway is considered as a tumor suppressor pathway due to its ability to impede cell proliferation and facilitate apoptosis (84). Similarly, NOTCH (85) and TP53 (86) pathway mutations have also been reported to associate with unfavorable prognosis in lung cancer. DNA damage response plays a significant role in maintaining genomic integrity and closely associated with lung cancer progression and treatment (87, 88). These researches provide additional insights into our observed outcomes that patients with HAS experience poorer survival when compared to those with LAS patients.

Cellular senescence functions as a stress response characterized by a halt in proliferation and heightened secretion of pro-inflammatory cytokines (89). Senescent cells recruit immune cells, facilitating their own immune clearance, thereby restoring tissue homeostasis. In the context of cancer, various stressors such as oncogenic signaling, replication stress, hypoxia, reactive oxygen species, nutrient deprivation, and exposure to cytokines within the tumor microenvironment can trigger senescence. This underscores the significant link between tumor cell senescence and immune cell infiltration. Through a bulk-transcriptome analysis, we observed that senescence-associated genes exert a strong influence on the immune microenvironment in LUAD. Specifically, the LAS group showed an activated TME, this manifested as a noteworthy increase in the quantities of CD8+ T cells, CD8+ Tcm, CD4+ Tem, CD4+ Tcm, plasma cells, mast cells and DC, alongside heightened ImmuneScore, GEP score and type II IFN response, T cell co-stimulation, and HLA scores, in addition to enriched immune response pathways. These findings were further corroborated though single-cell analysis, which revealed that CD8-CCR7 (T05), CD8-CXCL13 (T06), CD8-GNLY (T08), FCGR3A NK cells (T09), XCL1 NK cells (T10), plasma cell sets (B03, B04, B05), and mast cells (M01) were more enriched in the LAS group (**Figure 4**). In contrast, the HAS group displayed an immunosuppressive microenvironment with lower immune function scores and a higher tumor proliferation rate (**Figure 4**). Additionally, based on the cellular communication results, we identified some signaling pathways specific to the HAS group, such as SPP1 and TENASCIN (**Figure 4**), which contribute to tumor immune escape and tumor progression (50–52). These results suggest that the HAS group might promote tumor cell invasion by evading immune surveillance, enhancing proliferation and immune escape, leading to poor prognosis in LUAD.

In addition to bulk-level senescence assessment, we also compared senescence at the single-cell level and found significant heterogeneity in cellular senescence. Interestingly, we found that the age scores for tumor cells in the HAS group were significantly lower than that in the LAS group (**Figure 5C**), suggesting that senescence at the bulk-level is not the same as senescence at the cellular level. Senescent tumor cells might augment the immune response against tumors (90), which is entirely consistent with the highly senescent tumor cells and activated immune microenvironment in the LAS group. However, it’s worth noting that these senescent cells could also reinforce the tumor’s resistance to immunotherapy through potent immunosuppressive mechanisms (91, 92). Therefore, more in-depth studies at the cellular level remain essential.

Herein, we also explore the relationship between senescence and other modes of cell death. Patients in the HAS-group demonstrated a propensity for Alkaliptosis and ROS cell death mechanisms such as Oxeiptosis (93) and Cuproptosis. These endogenous damages, coupled with certain exogenous factors, induced a wide array of genetic injuries, including point mutations and deletions (94), ultimately leading to significantly higher TMB in the HAS-group compared to the LAS-group. To counteract DNA damage, the HAS-group employed a series of intricate DNA repair and maintenance mechanisms associated with cell proliferation and differentiation, ensuring the preservation of proper chromosomal structure and stability (8, 94). Conversely, the interactions among lysosome-dependent cell death, autophagy, and apoptosis played a more significant role in the LAS-group. Meanwhile, the LAS-group exhibited immunological functions in response to cellular senescence, engaging in tissue repair through immune cell recruitment and immune clearance of senescent cells.

More novel analyses were added to our study, although studies related to senescence in LUAD have been reported (15–18, 20). Firstly, although previous studies have also compared differences between aging subgroups in terms of mutations, or TMB (15–18). Patients with higher risk scores had noticeably increased TMB and mutated more frequently for TP53 (15, 16, 18), which is consistent with the results we found. Furthermore, our study was the first to compare at the pathway level which showed significant differences in patients with different ARRs. Second, existing researches related to senescence in LUAD have found that the lower risk scores group embodies an immune-activated microenvironment. Lin, et al., 2023 showed that the ASRS was positively correlated with most immunomodulator-related mRNAs, including chemokines, and immune inhibitors, and receptors (18). This study collected a previously reported set of 13 immune-related gene sets (41) and comprehensively compared the immunity of different subgroups. We found that nine of the 13 immune function gene sets were positively correlated with ARRS score, including APC to stimulation, cytokine and cytokine receptor (CCR), Check-point, cytolytic activity, inflammation-promoting, HLA, T cell co-stimulation, T cell co-stimulation, and type II IFN response (**Figure 3**). Thirdly, previous studies based on different datasets and different methods have been performed to show the association between immune infiltration and senescence. However, sometimes inconsistent results were obtained by different software. Our study evaluates the association between immune infiltration and senescence for the first time at the single cell level, and using scRNA-seq, this study compared cellular communication between different senescence groups, revealing possible alterations in cellular communication caused by senescence (**Figure 4**). Finally, we assessed senescence at the cellular level for the first time and found significant inter-cellular heterogeneity for senescence. In particular, we found an opposite trend between the overall senescence score and the tumor cell senescence score. This study still had some limitations, the limited availability of single-cell samples and immune cohort samples may introduce some bias in our model validation. Although we validated the aging score model using several external independent public datasets, prospective clinical trials verification of our model is still necessary. Nevertheless, we hope that this model can contribute to the comprehension of the molecular mechanisms of cellular senescence and TME in LUAD.

In conclusion, our study identified and validated a senescence-related signature based on 10 senescence-related genes as an independent prognostic significance for patients with LUAD, indicating that the senescence levels are heterogeneous in LUAD immune microenvironment, and the HAS group might promote tumor cell invasion by evading immune surveillance, enhancing proliferation and immune escape, leading to poor prognosis in LUAD.

## Data Availability

The datasets presented in this study can be found in online repositories. The names of the repository/repositories and accession number(s) can be found in the article.
