# Supplementary material for: Exploring the molecular and immune landscape of cellular senescence in lung adenocarcinoma
Source: Front Immunol. 2024 Aug 29;15:1347770. doi: 10.3389/fimmu.2024.1347770 (PMC11390420; doi:10.3389/fimmu.2024.1347770)
Supplement: Supplementary file 1 [file DataSheet1.docx]

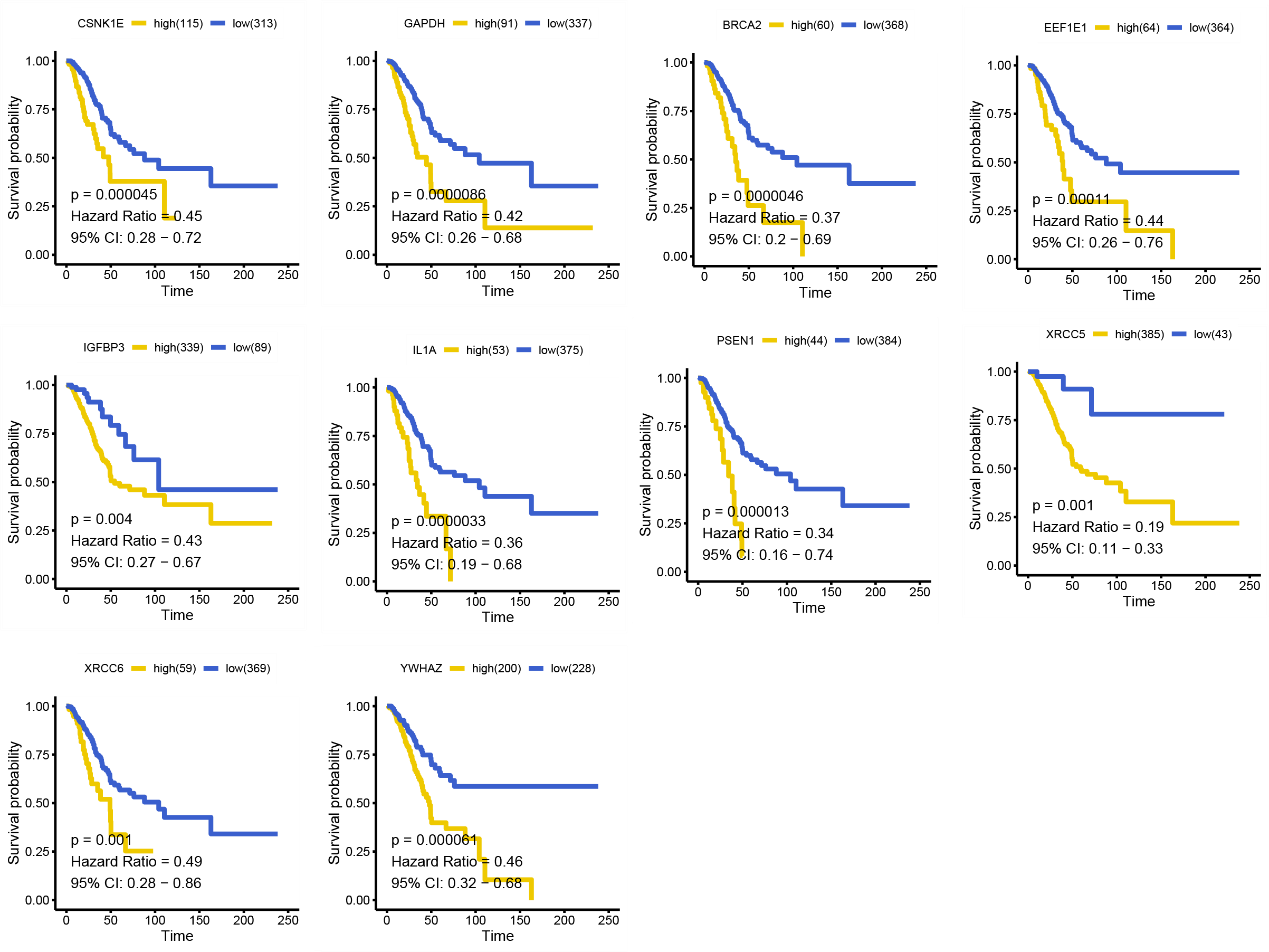


Supplementary Figure 1. Prognostic value of the ten ARKGs. Kaplan-Meier curves for overall survival in the TCGA-LUAD cohort. The optimal cut-off point of each gene was determined using the surv_cutpoint function in the survminer R package. Patients in the TCGA-LUAD cohort were divided into high and low expression groups according to the cut-off points. High expression of these genes was significantly associated with poor survival.


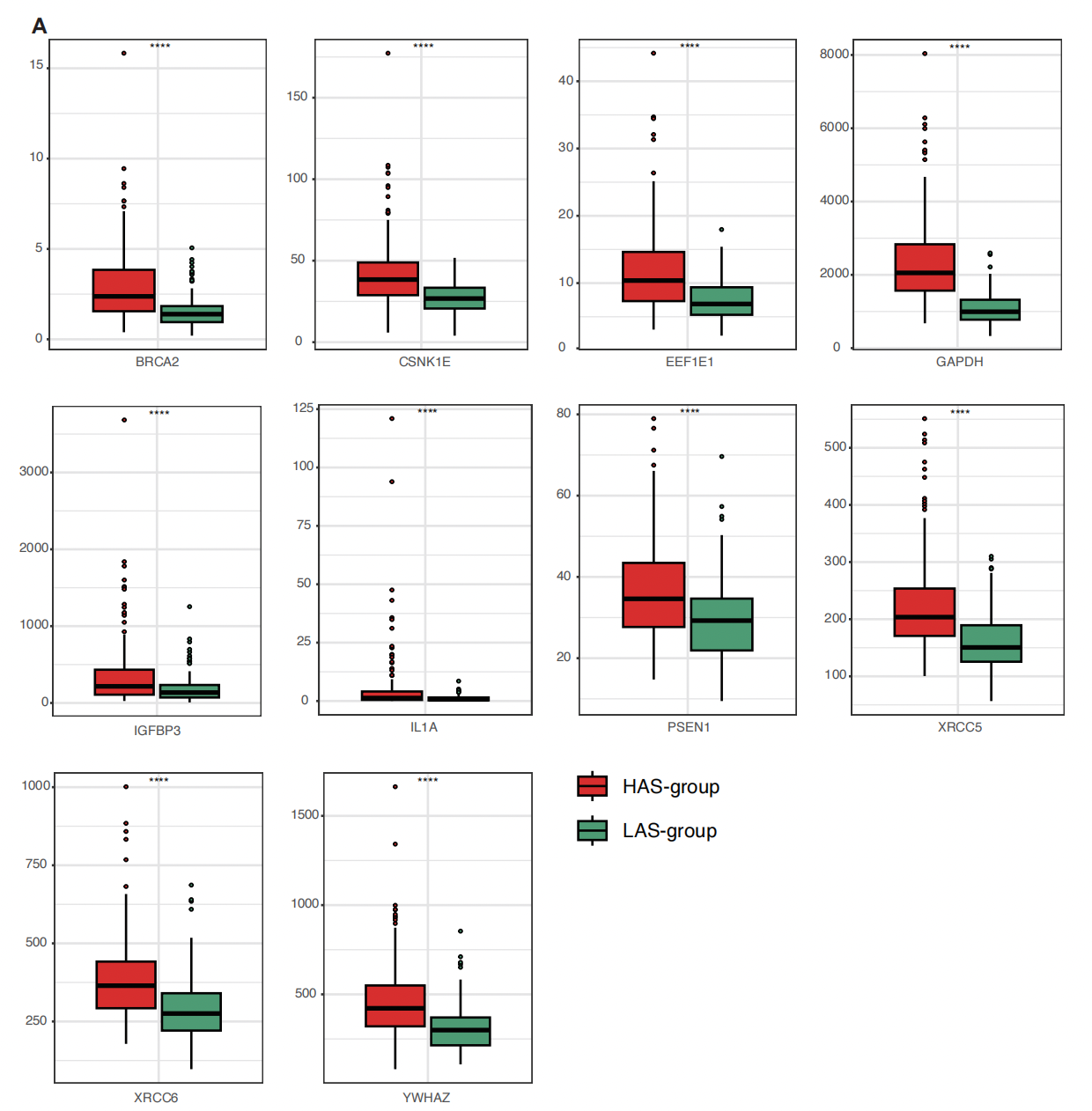


Supplementary Figure 2. Expression levels of the ten ARKGs.


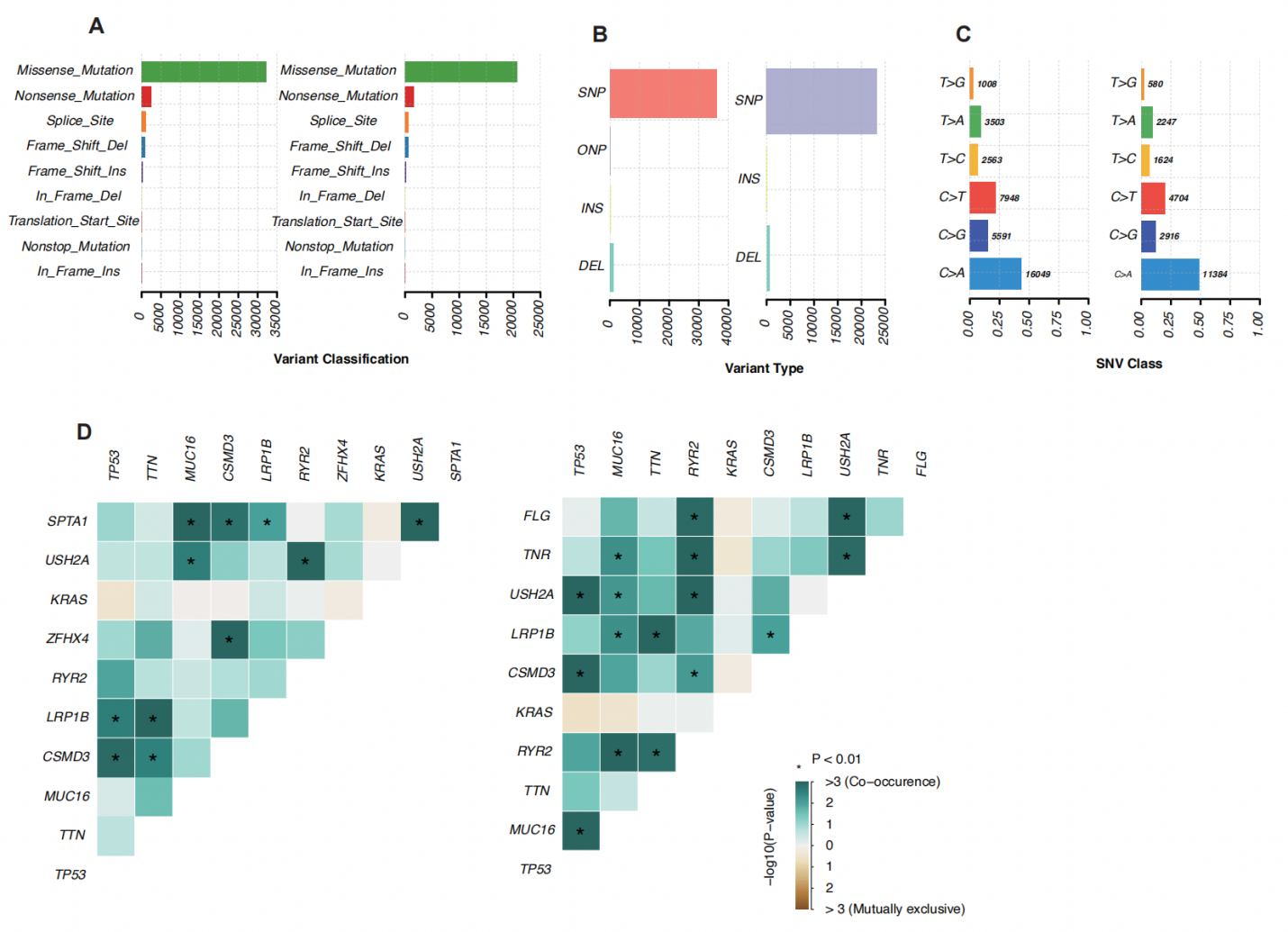


Supplementary Figure 3. Genomic alterations differences between the HAS and LAS group. (A)Variant classification landscape between the HAS (left) and LAS (right) group. (B) Variant type differences (B), SNV class (C) and co-occurring mutations (D) in HAS-group (left) compared to LAS-group (right).


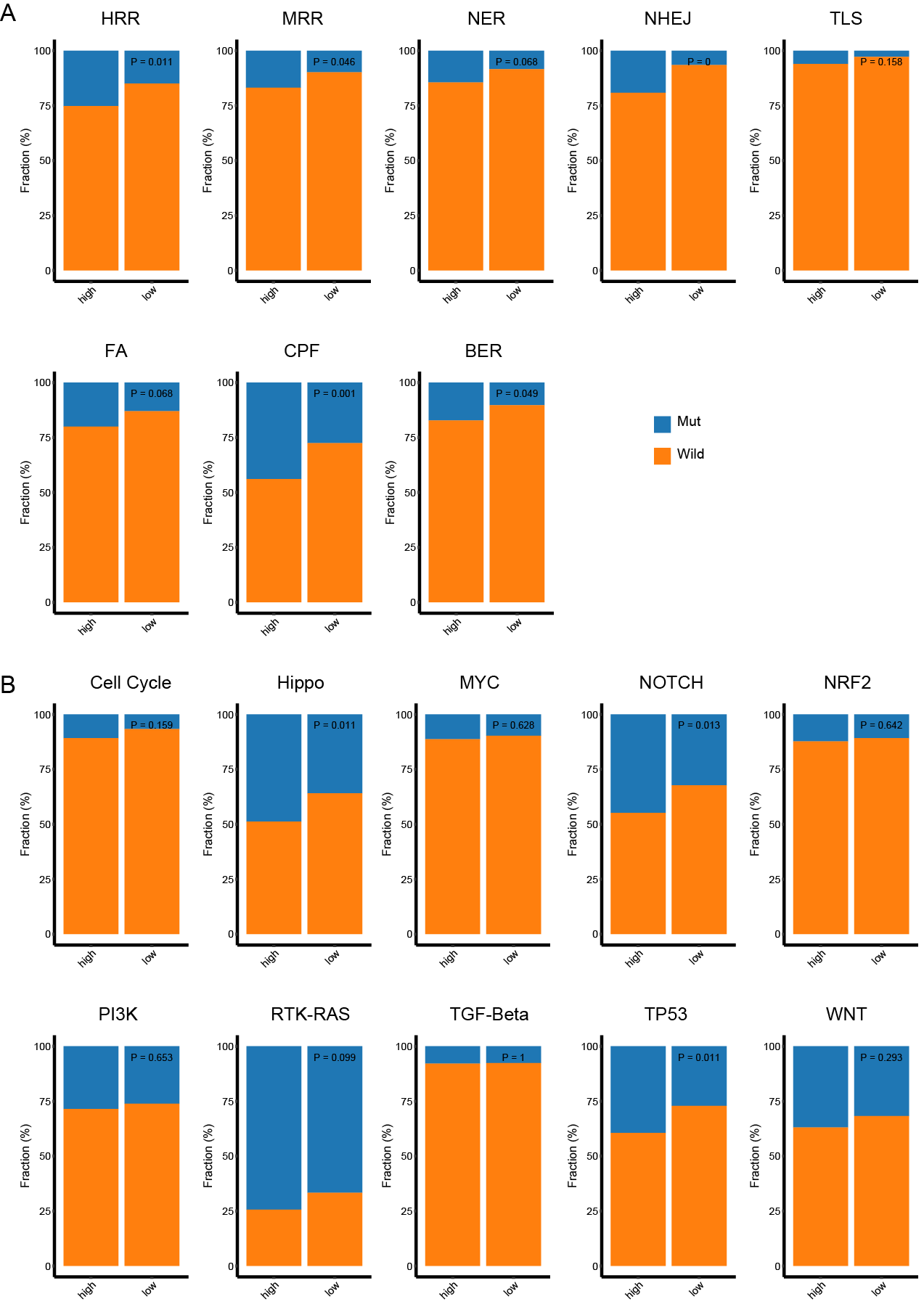


Supplementary Figure 4. The frequency of mutated genes in each tumor-related pathway (B) and DDR-related pathway (A) between HAS and LAS group.


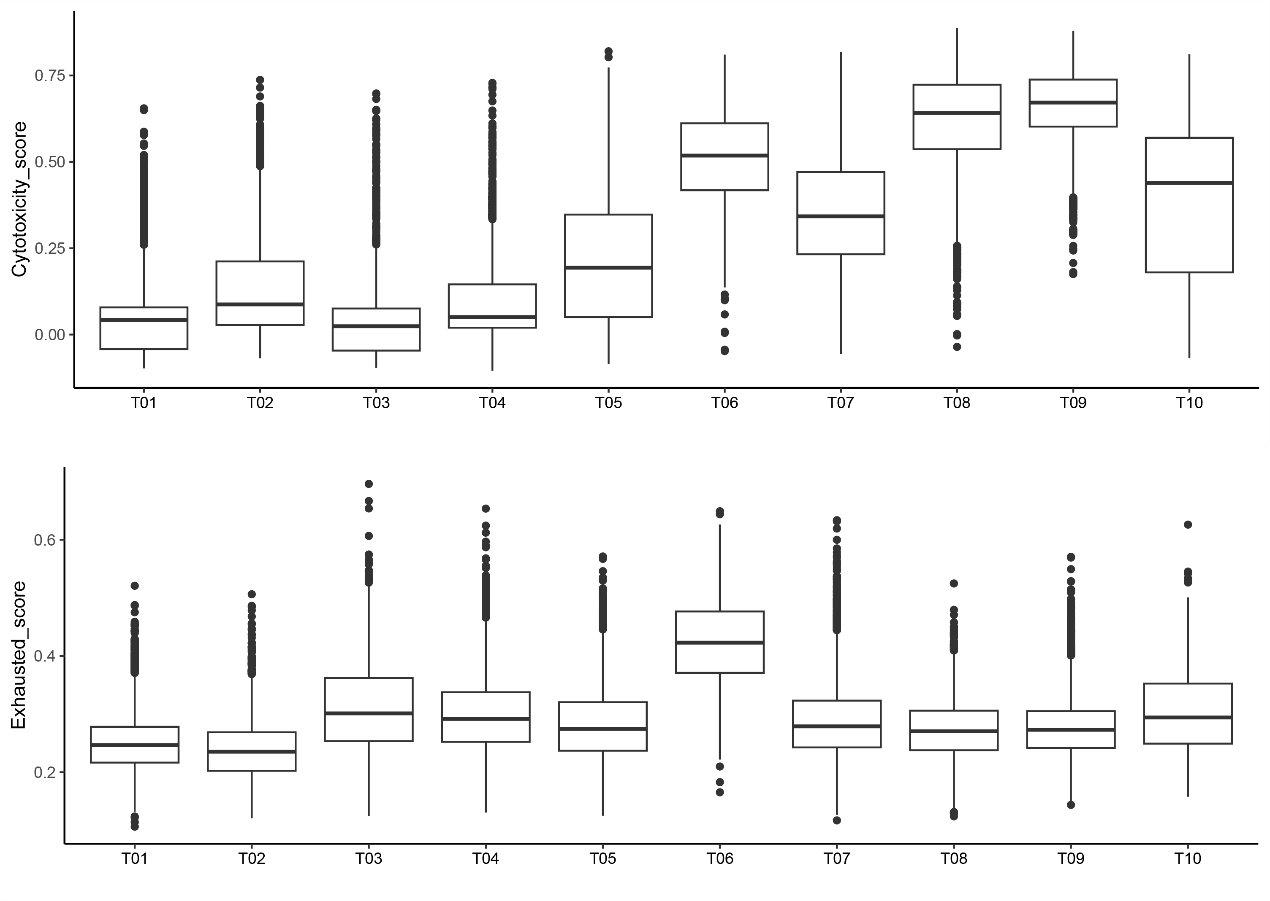


Supplementary Figure 5. The cytotoxic and exhausted scores for T-cell subsets.


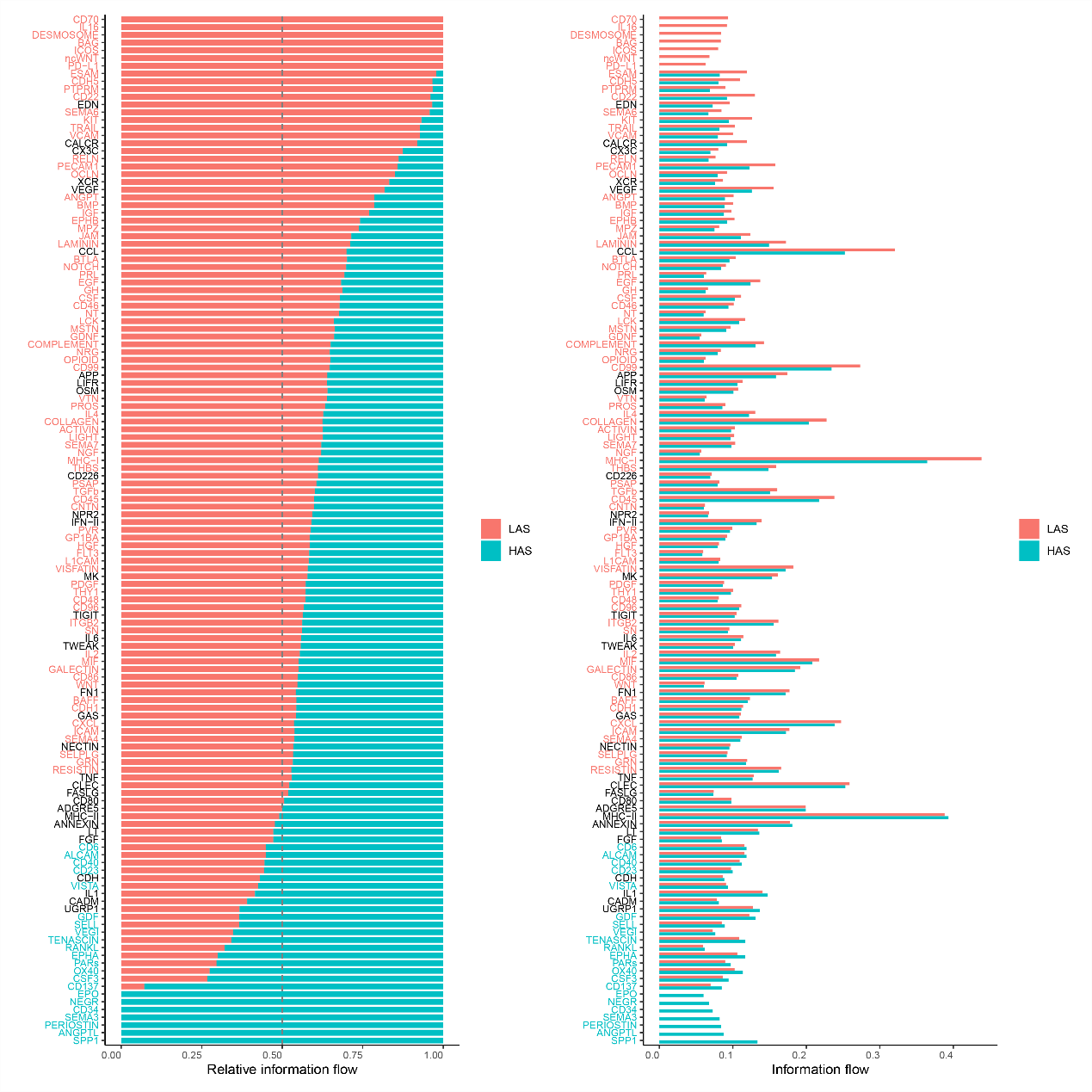


Supplementary Figure 6. Significant signaling pathways were ranked based on differences in the overall information flow within the inferred networks between HAS and LAS groups. The overall information flow of a signaling network is calculated by summarizing all communication probabilities in that network. The top signaling pathways colored red are enriched in LAS group, and signaling pathways colored green were enriched in the HAS group.
